# Tumor mutational burden is associated with poor outcomes in diffuse glioma

**DOI:** 10.1186/s12885-020-6658-1

**Published:** 2020-03-12

**Authors:** Lihong Wang, Jia Ge, Yang Lan, Yu Shi, Ying Luo, Yuhuan Tan, Mei Liang, Song Deng, Xia Zhang, Wenying Wang, Yaoyao Tan, Yuanyuan Xu, Tao Luo

**Affiliations:** 1 Institute of Pathology and Southwest Cancer Center, Southwest Hospital, Third Military Medical University (Army Medical University) and Key Laboratory of Tumor Immunopathology, Ministry of Education of China, Chongqing, 400038 China; 2 Bio-Bank of Southwest Hospital, Third Military Medical University (Army Medical University), Chongqing, 400038 China

**Keywords:** TMB, Glioma, Prognosis, Pan-cancer targeted sequencing

## Abstract

**Background:**

Tumor mutational burden (TMB) is a potential biomarker for immune checkpoint therapy and prognosis. The impact of TMB on clinical outcomes and the correlation coefficient between exome sequencing and targeted sequencing in glioma have not yet been explored.

**Methods:**

Somatic mutations in the coding regions of 897 primary gliomas and the clinical and RNA-seq data of 654 patients in The Cancer Genome Atlas (TCGA) database were analyzed as a training set, while another 286 patients in the Chinese Glioma Genome Atlas (CGGA) database were used for validation. Descriptive and correlational analyses were conducted with TMB. Enrichment map analysis and gene set enrichment analysis (GSEA) were also performed.

**Results:**

TMB was higher for the group of mutant genes that are frequently mutated in glioblastomas (GBMs) and lower for the group of mutant genes that are frequently mutated in lower-grade gliomas (LGGs). Patients with a higher TMB exhibited shorter overall survival. TMB was associated with grade, age, subtype and mutations affecting genomic structure. Moreover, univariate and multivariate analyses showed that TMB was an independent prognostic factor for glioma. The signaling pathways of the cell cycle were enriched in the TMB^High^ group. TMB was higher in the mismatch repair (MMR) gene mutant group than in the wild-type group, but the MMR pathway was enriched in the TMB^High^ group of gliomas without mutations in classical MMR genes. The correlation between TMBs calculated through exome sequencing and targeted sequencing was moderate, and panel-based TMB was not correlated with prognosis.

**Conclusions:**

TMB is associated with poor outcomes in diffuse glioma. The high proliferative activity in the TMB^High^ group could account for the shorter survival of these patients. This association was not reflected by a pan-cancer targeted sequencing panel.

## Background

Glioma is the most common malignant primary brain tumor in adults. Molecular classification via genomics, transcriptomics and methylomics has revealed the potential value of diagnosis based on molecules [1–3]. With the publication of the 2016 WHO classification, integrated diagnosis including mutational and histological phenotypes has been broadly applied in pathological typing. In some cases, the genotype even trumps the histological phenotype [4]. Tumor mutational burden (or tumor mutational load) is a potential biomarker of immune checkpoint inhibitors in many cancer types, as neoantigens are generated by somatic tumor mutations [5]. T cell-inflamed GEPs (gene expression profiles) are used to predict the response to PD-1 blockade and combined with TMB, they are used to predict the effects of anti-PD-1 treatment [6]. TMB is also a poor prognostic marker for neuroblastoma but a good prognostic marker for non-small-cell lung cancer [7, 8]. Furthermore, deficiency of the MMR complex leads to the accumulation of mutations [9, 10]. Exome sequencing or targeted sequencing is used to measure TMB. It is reported that panel-based TMB is highly correlated with TMB calculated by exome sequencing [9, 11]; thus, panel-based TMB is commonly used in cancer patients to predict survival after immunotherapy [5].

In glioma, the reports of TMB seem to be controversial. TMB is higher in LGG than in GBM [12]; on the other hand, the TMB of LGG is lower than that of GBM [13]. It has also been reported that the correlation of TMB and grade is not significant [14]. The prognostic value and related signaling pathways of TMB in glioma are still not known. In this study, using multi-omics data from TCGA, we systematically analyzed the correlations between TMB and mutational distribution, clinical features and transcriptomic data, revealing potential value for predicting prognosis and the related enrichment pathways of TMB in glioma. With CGGA data, we validated that TMB was an independent biomarker of prognosis. Furthermore, we found that MMR pathways were activated in high-TMB glioma patients without mutations in MMR genes. Finally, we evaluated the correlation between exome sequencing-based TMB and targeted sequencing-based TMB as well as the prognostic value of panel-based TMB, and the results indicated that it was inappropriate to predict TMB and prognosis with pan-cancer targeted sequencing in glioma.

## Methods

### Data source

The training set included the exome sequencing data (level 2, *n* = 897), RNA-seq data (*n* = 669) and clinical data (*n* = 1105) of patients with LGG and GBM from TCGA. Mutational data including variant allele frequencies of mutations were obtained from cBioPortal (http://www.cbioportal.org) [15, 16]. RNA-seq data were obtained from GlioVis (http://gliovis.bioinfo.cnio.es/) [17]. Clinical data were collected from GlioVis and cBioPortal. The whole-exome sequencing data (*n* = 286), mRNA-seq data (*n* = 1018) and indicated clinical data of the validation set were obtained from CGGA (http://www.cgga.org.cn/index.jsp). Integrated diagnosis was performed according to the World Health Organization (WHO) classification (2016).

### TMB (tumor mutational burden)

The size of the whole-exome genomic region has been defined as 36 Mb. The size of the pan-cancer panel genomic region has been defined as 1.06 Mb. For the estimation of the TMB of the training set, we used the same approach as was outlined in a recent study [9], i.e., counting all coding somatic base substitutions and indels in the targeted regions, including “stop_/start_lost/frameshift_/missense_/inframe_” alterations. The software used to estimate TMB was Personal Cancer Genome Reporter software [18]. The TMB of the validation set was calculated with somatic mutations (including single nucleotide variations and short insertions/deletions) identified by SAVI2 as previously described on the CGGA website (http://www.cgga.org.cn/about.jsp).

### Statistical analysis

The Mann-Whitney test was performed to compare the TMBs of two different groups. The Kruskal-Wallis test was used to compare the TMBs of more than two different groups. Spearman’s rank correlation test was used to examine the associations between TMB and age and gene expression. The optimal cut-off value was determined by X-tile. Patient survival was analyzed by the Kaplan-Meier method. The following covariates were used in Cox regression analysis (univariate and multivariable): TMB group, sex, WHO grade, histology, IDH status and chromosome 1p/19q codeletion. *p* < 0.05 was considered statistically significant (*p < 0.05, **p < 0.01, ***p < 0.001).

### GSEA and enrichment map

GSEA (gene set enrichment analysis) was performed with GSEA software (http://software.broadinstitute.org/gsea/downloads.jsp) [19], and GO biological process analysis including 4436 gene sets was performed (http://software.broadinstitute.org/gsea/msigdb/genesets.jsp?collection=BP). An enrichment map was used to visualize the results of GSEA according to previously reported methods [20].

## Results

### Mutational distribution according to the elevation in TMB

**Fig. 1:**
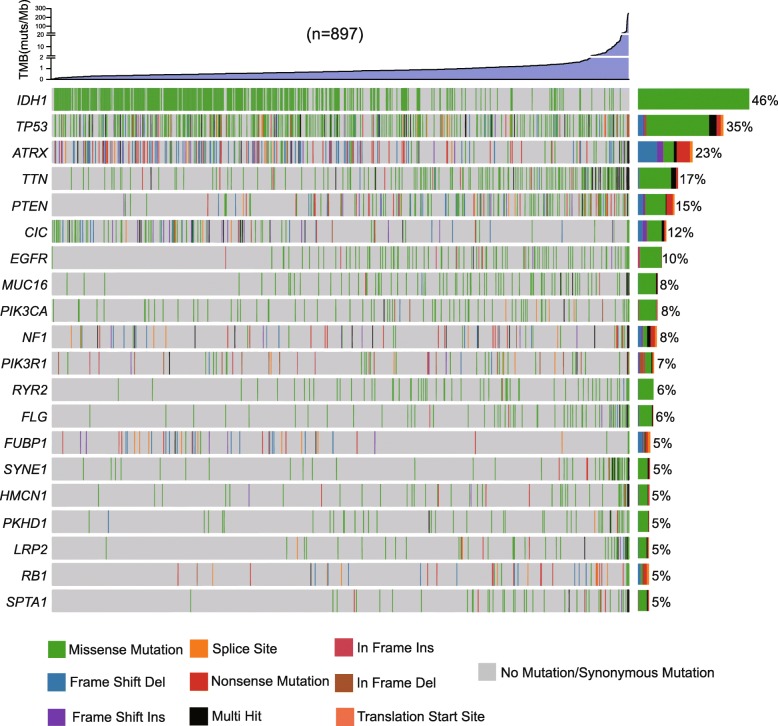
Heatmap showing the top 20 genes’ mutational frequencies and their types in glioma (*n* = 897)

To study the value of TMB in glioma, we first analyzed the types and distributions of nonsynonymous mutations (Supplementary Table 1). As previously reported, the mutation frequencies of *IDH1* were higher in LGG than in GBM, and the mutation rates of *PTEN and EGFR* were higher in GBM than in LGG. We confirmed this conclusion through the analysis of mutational frequencies in LGG and GBM (*IDH1*, 77% vs 7%; *PTEN*, 5% vs 29%; EGFR, 5% vs 17%; Supplementary Figs. 1 and 2). Tumors with elevated TMB are enriched for mutations in PTEN or EGFR. Consistently, IDH mutations were primarily found in tumors with low TMB (Fig. 1). We further analyzed the statistical significance of TMB in the mutant and wild-type groups of the 20 genes. With correction for multiple hypotheses, we found that TMB was higher in the *PTEN* mutant group than in the *PTEN* wild-type group but lower in the *IDH1* mutant group than in the *IDH1* wild-type group (Table 1).

**Table 1:** Statistical significance of TMB in mutant and wildtype group of indicated genes in gliomas(*n* = 897)

	Number of mutant group	Number of wildtype group	Median TMB of mutant group	Median TMB of wildtype group	*P* value (Mann-Whitney test)	False discovery rate (FDR)	Adjust *p*-value (bonferroni)
IDH1	413	484	0.53	0.97	2.3E-58	4.6E-57	4.6E-57
TP53	317	580	0.67	0.81	5.5E-02	5.5E-02	1.0E+ 00
ATRX	203	694	0.61	0.81	1.2E-05	1.6E-05	2.4E-04
TTN	149	748	1.11	0.69	3.9E-20	3.9E-19	7.9E-19
PTEN	136	761	1.045	0.67	1.7E-18	1.2E-17	3.5E-17
CIC	106	791	0.47	0.78	1.4E-11	3.4E-11	2.7E-10
EGFR	89	808	1.08	0.69	2.1E-14	1.1E-13	4.3E-13
MUC16	74	823	1.06	0.72	5.6E-10	1.0E-09	1.1E-08
PIK3CA	74	823	0.92	0.72	1.4E-03	1.8E-03	2.9E-02
NF1	70	827	0.9	0.72	1.2E-02	1.4E-02	2.4E-01
PIK3R1	60	837	0.89	0.72	2.7E-02	2.9E-02	5.5E-01
RYR2	58	839	0.985	0.72	2.7E-02	2.9E-02	5.5E-01
FLG	57	840	1.33	0.72	1.8E-13	5.9E-13	3.6E-12
FUBP1	46	851	0.5	0.75	1.8E-13	5.9E-13	3.6E-12
SYNE1	45	852	4	0.72	6.8E-11	1.5E-10	1.4E-09
HMCN1	43	854	1.08	0.72	2.0E-08	3.0E-08	3.9E-07
PKHD1	42	855	1.14	0.72	2.6E-07	3.7E-07	5.2E-06
LRP2	41	856	1.25	0.72	1.2E-10	2.4E-10	2.4E-09
RB1	41	856	1.22	0.72	9.9E-09	1.7E-08	2.0E-07
SPTA1	41	856	1.25	0.72	3.0E-12	8.7E-12	6.1E-11

**Table 2:** Univariate and multivariable Cox regression analyses of factors associated with overall survival in glioma patients

Variable	Univariate analysis		Multivariable analysis	
	HR (95% CI)	P^*^	HR (95% CI)	P
Age	1.86(1.19 to 2.90)	< 0.01	/	/
Gender	1.31(0.87 to 1.98)	0.19	/	/
Grade	2.61(1.95 to 3.49)	< 0.01	2.08(1.53 to 2.82)	< 0.01
Histology	1.45(1.12 to 1.86)	< 0.01	/	/
IDH.status	0.36(0.24 to 0.54)	< 0.01	/	/
Chr.1p/19q.codeletion	0.12(0.05 to 0.27)	< 0.01	0.15(0.06 to 0.36)	< 0.01
TMB group	2.58(1.67 to 3.97)	< 0.01	1.90(1.21 to 2.98)	< 0.01

^*^All statistical tests were two-sided. *CI* confidence interval, *HR* hazard ratio, *TMB* cut-off value = 0.655 mutations/Mb

### TMB is associated with worse outcomes in glioma patients

**Fig. 2:**
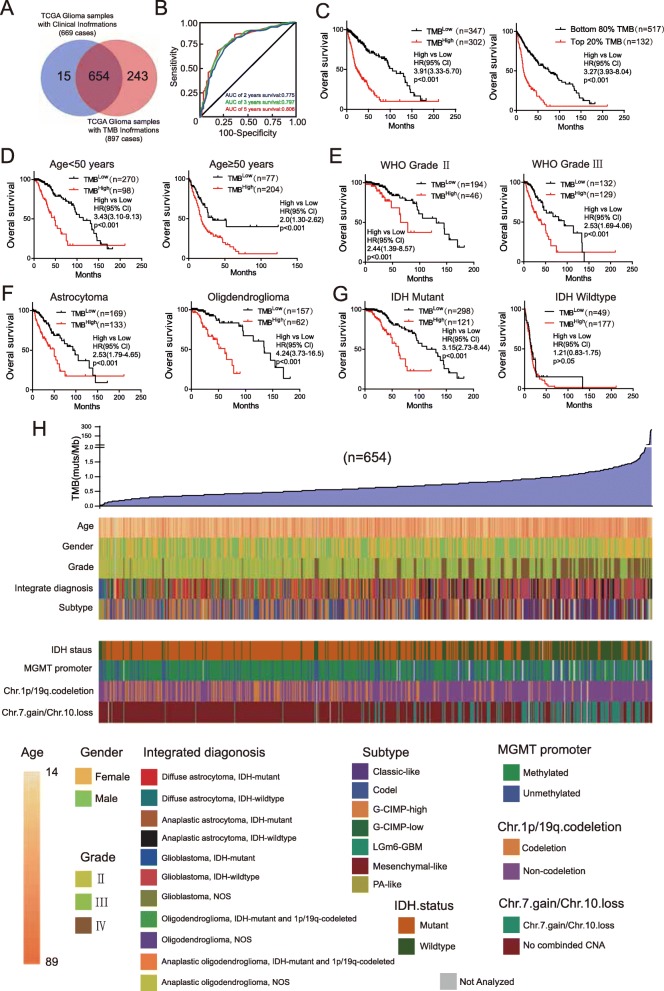
TMB is associated with worse outcomes in glioma patients. **a** Venn diagram of the patients included in further analysis. **b** ROC analysis of 2-, 3-, and 5-year survival according to TMB. **c** Kaplan–Meier curves of the overall survival of glioma patients (*n* = 649, 5 patients lacked survival information) with high TMB (TMB^High^) versus those with low TMB (TMB^Low^). The cut-off value of the left panel was between 0.64 and 0.67. The right panel had the highest 20%. **d-g** Survival analysis was performed in the indicated subgroups. **h** Heatmap showing the distribution of clinical features and genetic characteristics of the glioma specimens (*n* = 654)

We further analyzed the relationships between TMB and clinical features (Supplementary Table 2, *n* = 654). Patients without clinical or mutational information were excluded (Fig. 2a). As we expected, TMB increased according to grade (median TMB, 0.47 vs 0.64 vs 0.99 mutations/Mb; Supplementary Fig. 3A). Through ROC analysis, the AUCs for TMB for 2-, 3-, and 5-year survival were 0.775, 0.797, and 0.806, respectively (Fig. 2b). We determined the cut-off value (between 0.64 and 0.67 mutations/Mb) of TMB with X-tile software, and the patients were divided into TMB^High^ and TMB^Low^ groups. Overall survival was decreased in patients with a high TMB compared to those with a low TMB (hazard ratio 3.91, 95% confidence interval 3.33–5.70; *p* < 0.001, log-rank; Fig. 2c, left). Patients in the TMB^High^ group exhibited a median overall survival of 23.0 months, whereas those in the TMB^Low^ group exhibited a median overall survival of 105.2 months. We confirmed the prognostic effect of TMB with the top 20% of patients as TMB^High^ group and the bottom 80% of patients as TMB^Low^ group (hazard ratio 3.27, 95% confidence interval 3.93–8.04; *p* < 0.001, log-rank; Fig. 2c, right). To determine the prognostic value in the context of established risk factors, we performed survival analysis in different subgroups (Fig. 2d-g). TMB was significantly associated with poor outcomes in the indicated subgroups except for the glioblastoma, WHO grade IV and IDH wildtype subgroups. To test whether TMB was an independent biomarker for prognosis, we performed Cox regression analysis in an independent validation set. In the univariate analysis, age, grade, histology, IDH status, chromosome 1p/19q codeletion and TMB were statistically significantly associated with overall survival (*p* < 0.01) (Table 1, Supplementary Table 3). In the multivariable analysis, grade, chromosome 1p/19q codeletion and TMB were independently associated with overall survival (Table 2). Furthermore, we analyzed the distribution of clinical features accompanied by an elevated TMB (Fig. 2g). TMB was significantly increased in older patients but was not associated with sex (Supplementary Fig. 3B, C). In the subgroup analysis of integrated diagnosis, TMB was found to be elevated in the anaplastic astrocytoma *IDH* wild-type group compared to the other astrocytoma group (Supplementary Fig. 3D). TMB was also increased for the classic-like and mesenchymal-like subtypes compared to other *IDH* wild-type subtypes and for the G-CIMP-low subtype compared to other *IDH* mutant subtypes (Supplementary Fig. 3E) [5]. Mutational analysis revealed that the patients exhibiting an unmethylated *MGMT* promoter, non-codeletion of 1p/19q and Chr.7.gain/Chr.10.loss exhibited a higher TMB (Supplementary Fig. 3F). Overall, these data indicated that TMB could be an independent prognostic biomarker of glioma.

### TMB^High^ gliomas exhibit elevated proliferative activity and immune responses

**Fig. 3:**
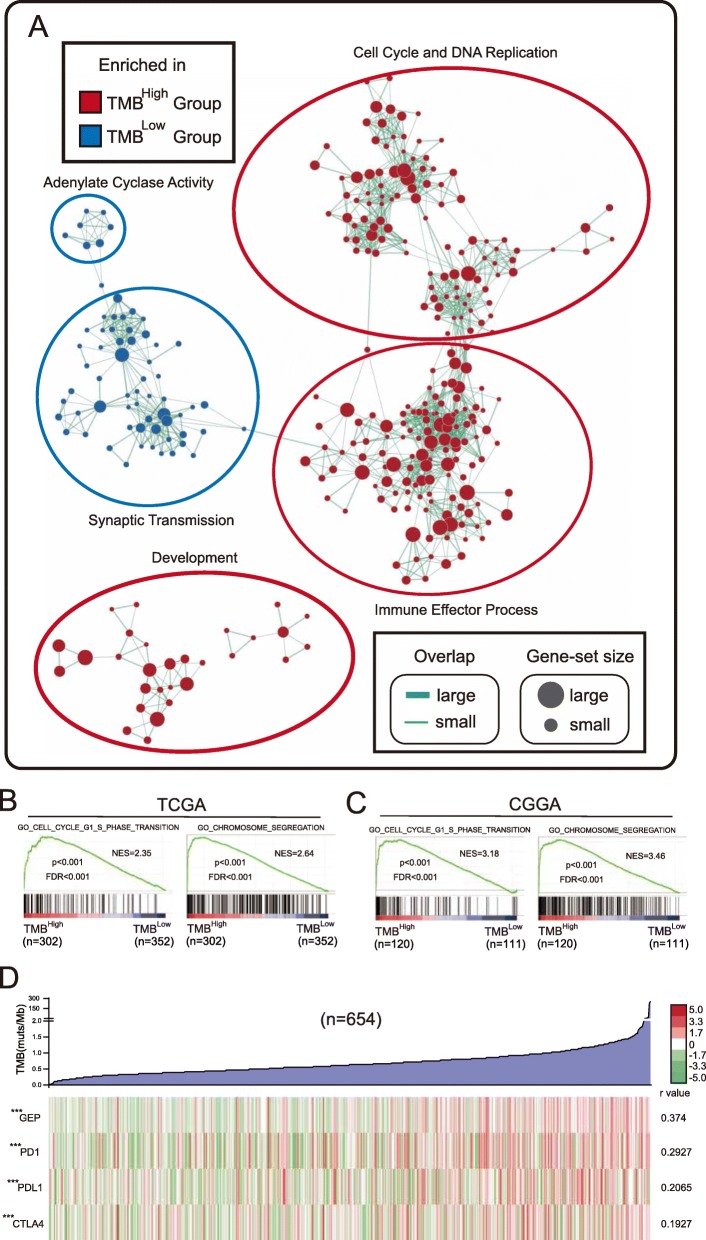
TMB^High^ gliomas exhibit increased proliferative activity and immune responses. **a** GO biological progress enriched by GSEA in the TMB^High^ group (*n* = 302) versus the TMB^Low^ group (*n* = 352) using an enrichment map. Node size represents the number of genes in the gene sets. Line width represents the number of overlapping genes. **b** Representative GSEA enrichment plots in (**a**). The NES (normalized enrichment score), *p* value and FDR (false discovery rate) were calculated with GSEA software. **c** GSEA of the validation set (CGGA) in the indicated gene sets. **d** The heatmap showing the distribution and correlation of the indicated gene set/genes in glioma specimens was visualized using Java Tree-view. Spearman’s r value and significance were calculated

To clarify the mechanism of the association between the TMB and poor outcomes of glioma patients, we analyzed the data from patients with TMB information and RNA-seq data (*n* = 654). Gene set enrichment analysis (GSEA) coupled with enrichment map analysis was performed to visualize the enriched biological processes. The TMB^High^ group was enriched in transcriptional programs related to the cell cycle, DNA replication and immune effector processes. In contrast, the transcriptional programs of adenylate cyclase activity and synaptic transmission were enriched in the TMB^Low^ group (Fig. 3a, b). These transcriptomics data indicated that high-TMB gliomas exhibit intensive proliferative activity, which might result in a worse prognosis. We performed GSEA in different subgroups and found that the TMB^High^ group was enriched in transcriptional programs related to the cell cycle when we controlled for age, WHO grade, histology and *IDH* status (Supplementary Fig. 4). GSEA of the validation set also confirmed the results (Fig. 3c). Furthermore, TMB exhibited a modest correlation with the inflammatory biomarkers of checkpoint inhibitor-based immunotherapy (Fig. 3d), which was consistent with the findings of previous reports based on the pan-cancer dataset [6].

### High TMB is associated with the mismatch repair pathway in gliomas without mutations in classical MMR genes

**Fig. 4:**
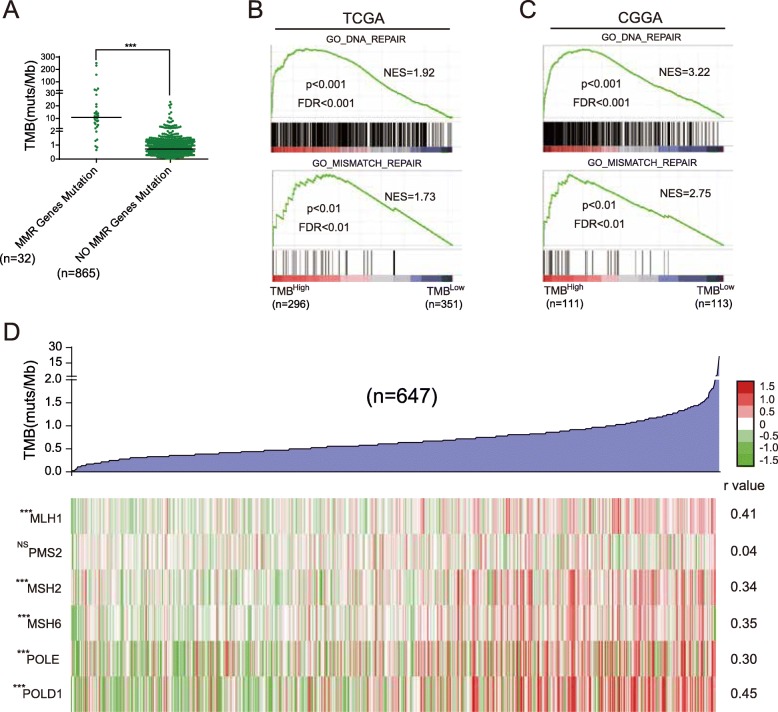
The mismatch repair pathway is activated in the TMB^High^ group in gliomas without mutations in classical MMR genes. **a** The TMB of gliomas with/without mutations in 6 classical MMR genes. **b-c** DNA repair and mismatch repair functions were analyzed by GSEA in the TMB^High^ group versus the TMB^Low^ group in the TCGA (**b**) and CGGA (**c**) datasets. **d** Heatmap analysis of the distribution and correlation of the indicated genes was performed. Spearman’s r value and significance were calculated

It has been reported that MMR (mismatch repair) deficiency is associated with a higher TMB in gliomas [14], and we confirmed this finding in the TCGA dataset. Only 3.6% of glioma patients harbored MMR gene mutations (32 of 897 glioma patients). TMB was elevated in patients exhibiting *MLH1*, *MSH2*, *MSH6*, *PMS2*, *POLD1* or *POLE* gene mutations (Fig. 4a). We further performed GSEA in patients without mutations in MMR genes to confirm whether high TMB was associated with low mismatch repair function. Interestingly, mismatch repair-associated transcriptional programs were also enriched in the TMB^High^ group but not in the TMB^Low^ group (Fig. 4b, c). The correlation analysis of TMB and the expression of MMR genes further demonstrated that a high TMB was associated with the expression of *MLH1*, *MSH2*, *MSH6*, *POLD1* and *POLE* in gliomas without MMR mutations (Fig. 4d). These data indicated that when the MMR genes are not mutated, TMB exhibits a positive correlation with MMR function.

### Pan-cancer targeted sequencing cannot predict prognosis in glioma

**Fig. 5:**
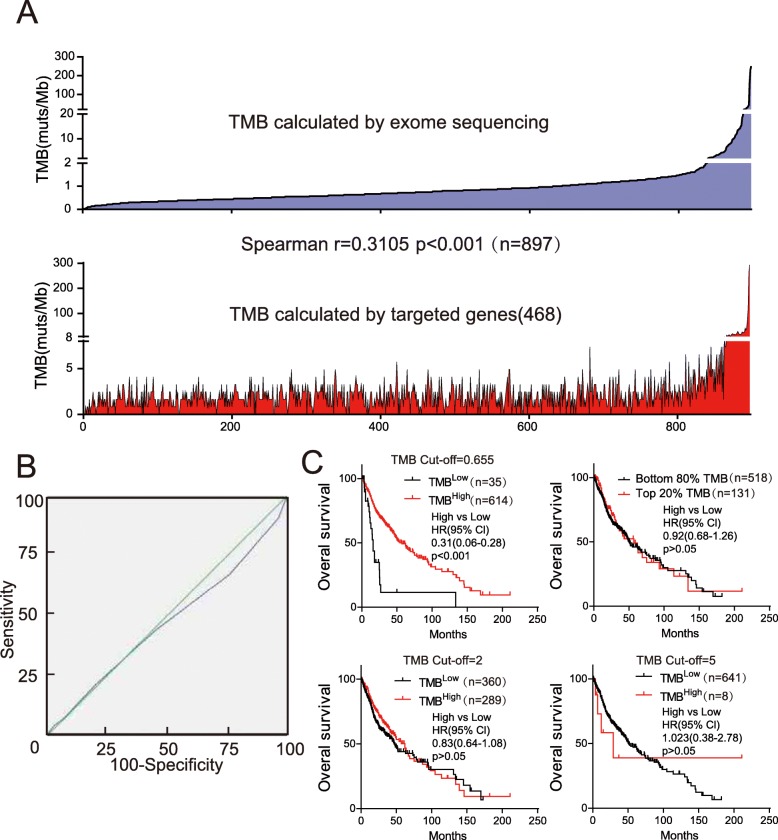
Pan-cancer targeted sequencing-based TMB cannot predict prognosis in glioma. **a** Correlation of TMB calculated on the basis of exome sequencing and targeted genes. The list of genes is from the FDA-approved targeted next generation sequencing panel (MSK-IMPACT). Spearman’s r value and significance were calculated. **b** AUC analysis with TMB calculated using the MSK-IMPACT panel. **c** Survival analysis was performed with the indicated cut-off value

Considering the cost of exome sequencing, targeted sequencing is widely used to predict TMB in pan-cancer analyses. We calculated TMB using 468 genes (Supplementary Table 4) from MSK-IMPACT [11] and analyzed the Spearman correlation with TMB calculated on the basis of exome sequencing (Fig. 5a, Supplementary Table 5). Interestingly, unlike other cancer types, the correlation between panel-based TMB and exome sequencing-based TMB was moderate in glioma (*r* = 0.3105). This result was confirmed with two other panels (Supplementary Table 4) that are used in China (Supplementary Fig. 5, *r* = 0.2753/0.3461). We further performed ROC analysis for panel-based TMB (Fig. 5b), and the result was significantly different from the ROC of exome sequencing-based TMB (Fig. 2b). We performed survival analysis with different TMB cut-off values. Only when the cut-off value was between 0.64 and 0.67 was TMB negatively associated with overall survival (Fig. 5c), which was not consistent with previous results (Fig. 2c). With the other cut-off value, TMB was not correlated with overall survival (Fig. 5c). These data indicated that, at least in glioma, pan-cancer panel-based TMB cannot represent exome sequencing-based TMB and is not suitable for prognosis.

## Discussion

In this study, to understand the value of TMB in glioma, we first analyzed the relationship between TMB and the mutation distribution. For most genes, TMB was elevated in the individual gene mutant group compared to the individual gene wild-type group. For example, TMB was higher in the *PTEN* mutant group than in the *PTEN* wild-type group. It is reasonable as TMB is an aggregate of mutations that result in protein alterations. However, for genes that were mutated in lower-grade glioma, such as *IDH1*, TMB was lower in the *IDH1* mutant group than in the *IDH1* wild-type group. Through the analysis of clinicopathological parameters, we found that TMB was significantly elevated with an increasing grade and was associated with the poor survival of patients. These results were validated with the CGGA dataset through multivariable Cox regression analysis. According to enrichment map analysis with GSEA, the TMB^High^ group exhibited activation of cell proliferation. As reported in pan-cancer studies, the correlation between TMB and T cell immunity was moderate, and TMB was elevated in the MMR gene mutant group. Interestingly, in the MMR gene wild-type group, the transcriptional programs of MMR enriched in the TMB^High^ group and TMB were positively correlated with the expression of MMR genes. Finally, by analyzing pan-cancer targeted sequencing, it was found that TMB based on panel analysis was not highly correlated with TMB calculated by exome sequencing in glioma. No prognostic value existed for targeted sequencing-based TMB.

The limitations of this study include optimal cut-off value and experimental validation. The conclusion could be totally different with different cut-off values. To address this concern, we analyzed the relative risk with different cut-off values by X-tile software. The optimal cut-off value was between 0.64 and 0.67, and we further tested the prognostic value with an independent dataset by multivariable analysis. GSEA is a promising method to explore the relationship between risk factors and signaling pathways with expression profiling data; however, causal links still need experimental validation. Although we confirmed the activation of cell proliferation in the TMB^High^ group with the CGGA dataset, it is essential to test this conclusion with primary glioma cells in vitro and in vivo.

With the development of large-scale sequencing, thousands of somatic mutations have been revealed in cancer samples. Different cancer types exhibit distinct mutational signatures [12]. The mutational landscape is reported to illustrate driver mutations and can be used to develop individualized treatments [21]. In glioma, the reported TMB is paradoxical. The mutational load calculated by exome sequencing is associated with the tumor grade and age of patients [22]. However, in another cohort, targeted sequencing-based TMB was not correlated with grade [14]. To study the application of targeted sequencing in the prediction of TMB in glioma, we analyzed the correlation between exome sequencing-based TMB and targeted sequencing-based TMB in a TCGA cohort. Although the correlation was significant, the coefficient was modest. For most gliomas with a TMB < 2, the targeted sequencing-based calculation could not exactly predict TMB. Although targeted sequencing is more economical than exome sequencing, pan-cancer targeted sequencing-based TMB may be inappropriate for predicting the prognosis of glioma patients. Glioma-customized panels should be designed to precisely predict the mutational load.

Emerging data imply that neoantigens resulting from nonsynonymous mutations could serve as potential biomarkers for checkpoint blockade therapy. The mutation that results in tumor initiation could also be targeted by the immune system [15]. In a mouse model, radiation plus anti-PD-1 treatments improved survival compared to radiation alone [23]. With immune checkpoint inhibitor treatment, the tumor size of glioblastomas with hypermutation was significantly reduced [24]. However, the failure of CheckMate-143 indicates that nivolumab does not improve the OS of patients with recurrent glioblastoma compared to bevacizumab treatment [25]. These data imply that the mutational state should be analyzed before immune checkpoint inhibitor treatment. We analyzed the relationship between TMB and other biomarkers of immune checkpoint inhibitors and illustrated the possible mechanism by which TMB is associated with poor survival. Our work revealed potential biomarkers for improving survival in glioma patients. However, our data were mainly obtained from patients treated with routine chemoradiotherapy. TMB should be tested in patients treated with immune checkpoint inhibitors.

## Conclusions

1. TMB is associated with shorter overall survival in glioma patients.

2. Proliferative activity and the immune response are activated in TMB^High^ gliomas.

3. TMB was higher in the MMR gene mutant group, but the MMR pathway was enriched in the TMB^High^ group.

4. Pan-cancer targeted sequencing-based TMB cannot predict prognosis in glioma.

## Supplementary information

**Additional file 1.**

**Additional file 2: Supplementary Figure 1.** The top 20 genes’ mutational frequencies and their types in LGG (*n* = 505).

**Additional file 3: Supplementary Figure 2.** The top 20 genes’ mutational frequencies and their types in GBM (*n* = 392).

**Additional file 4.**

**Additional file 5: Supplementary Figure 3.** TMB was associated with different grades (A), ages (B), sexes (C), integrated diagnoses (D), subtypes (E) and mutational statuses (F) in glioma. Statistical significance was calculated with the Mann-Whitney test for two groups and with the Kruskal-Wallis test for more than two groups.

**Additional file 6.**

**Additional file 7: Supplementary Figure 4.** The transcriptional programs of the cell cycle were enriched in the TMB^Low^ group at different ages (A), WHO grades (B), histology (C) and IDH statuses (D). The NES (normalized enrichment score), *p* value and FDR (false discovery rate) were calculated with GSEA software.

**Additional file 8.**

**Additional file 9.**

**Additional file 10: Supplementary Figure 5.** The correlation of TMB calculated on the basis of exome sequencing and targeted genes. Gene list from two local panels used in pan-cancer analysis. Spearman’s r value and significance were calculated.

## Data Availability

The datasets generated and/or analyzed during the current study are available in the TCGA (http://www.cbioportal.org), GlioVis (http://gliovis.bioinfo.cnio.es/) and CGGA (http://gliovis.bioinfo.cnio.es/) databases.

## Abbreviations

BP: Biological process
CGGA: Chinese Glioma Genome Atlas
GBMs: Glioblastomas
GEPs: Gene expression profiles
GO: Gene Ontology
GSEA: Gene set enrichment analysis
*IDH*: Isocitrate dehydrogenase
LGGs: Lower-grade gliomas
*MLH1*: MutL Homolog 1
MMR: Mismatch repair
*MSH2*: MutS Homolog 2
*MSH6*: MutS Homolog 6
MSK-IMPACT: Memorial Sloan-Kettering Cancer Center-Integrated Mutation Profiling of Actionable Cancer Targets
OS: Overall survival
*PMS2*: PMS1 Homolog 2, Mismatch Repair System Component
*POLD1*: DNA Polymerase Delta 1, Catalytic Subunit
*POLE*: DNA Polymerase Epsilon, Catalytic Subunit
*PTEN*: Phosphate and tension homology deleted on chromosome ten
ROC: Receiver operating characteristic
TCGA: The Cancer Genome Atlas
TMB: Tumor mutational burden
WHO: World Health Organization
