# Supplementary figures and images for: Tumor mutational burden is associated with poor outcomes in diffuse glioma
Source: BMC Cancer. 2020 Mar 12;20:213. doi: 10.1186/s12885-020-6658-1 (PMC7069200; doi:10.1186/s12885-020-6658-1)

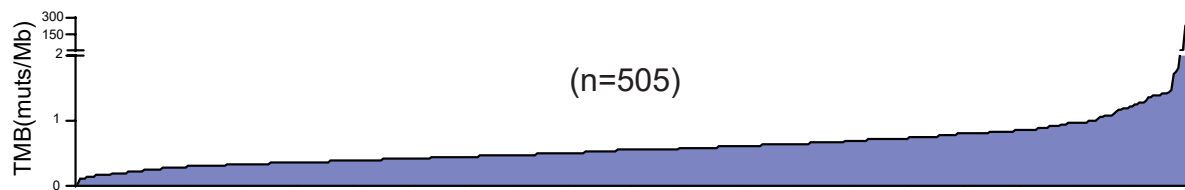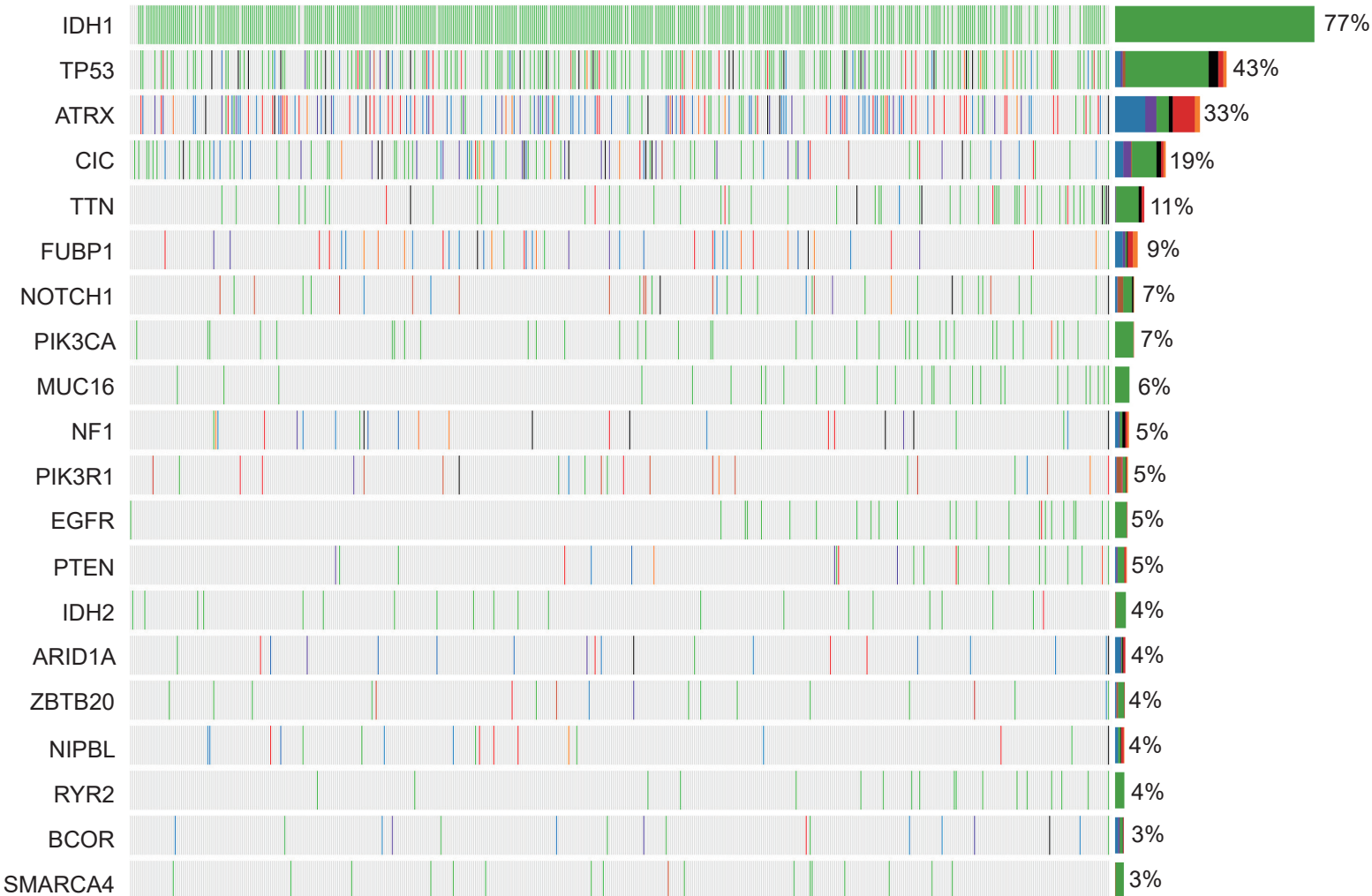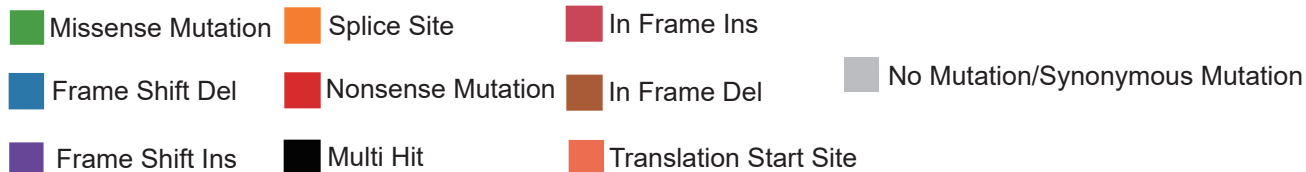

Supplement: Supplementary file 2 — Additional file 2: Supplementary Figure 1. The top 20 genes’ mutational frequencies and their types in LGG (n = 505). [file 12885_2020_6658_MOESM2_ESM.pdf]

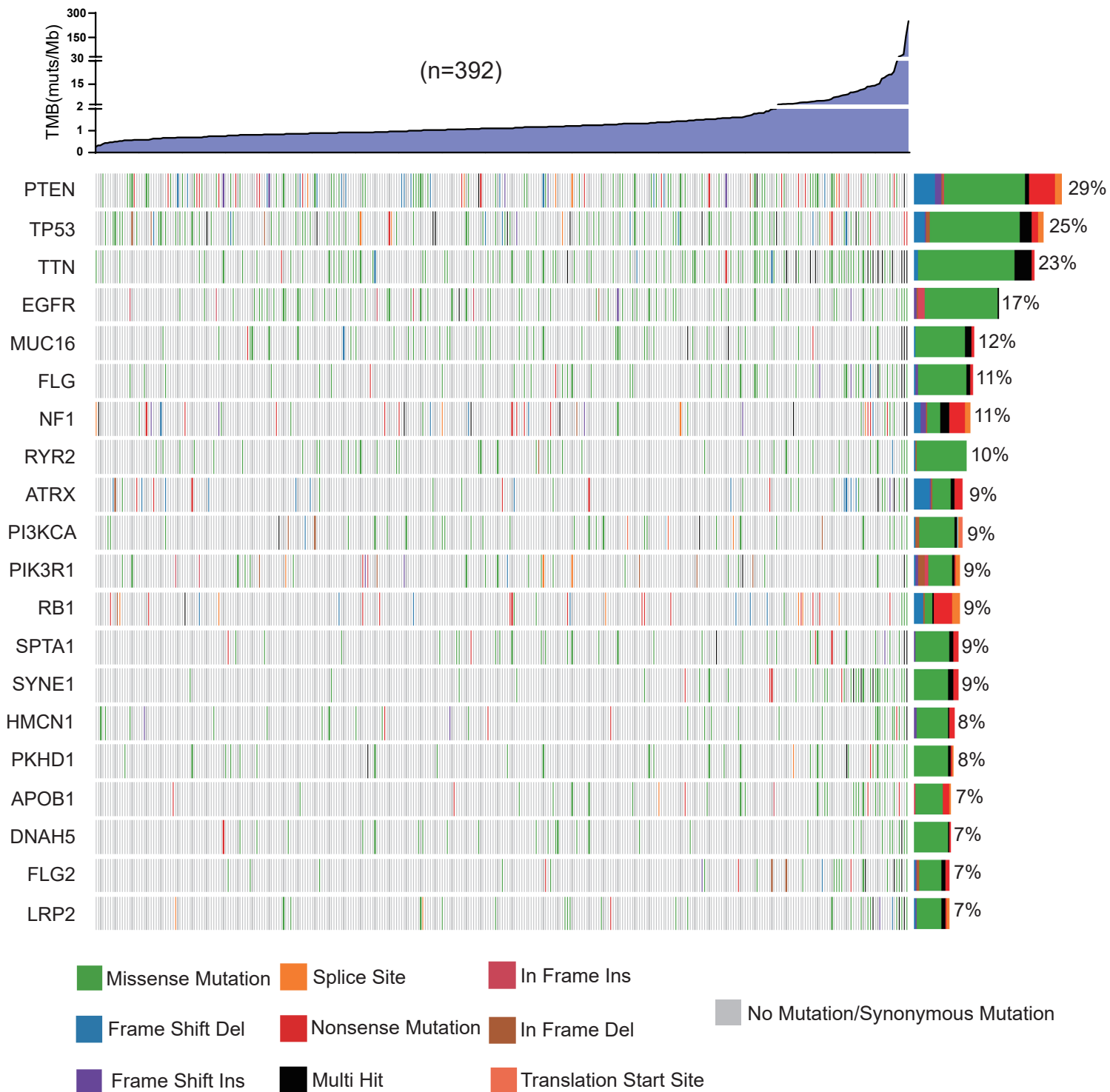

Supplement: Supplementary file 3 — Additional file 3: Supplementary Figure 2. The top 20 genes’ mutational frequencies and their types in GBM (n = 392). [file 12885_2020_6658_MOESM3_ESM.pdf]

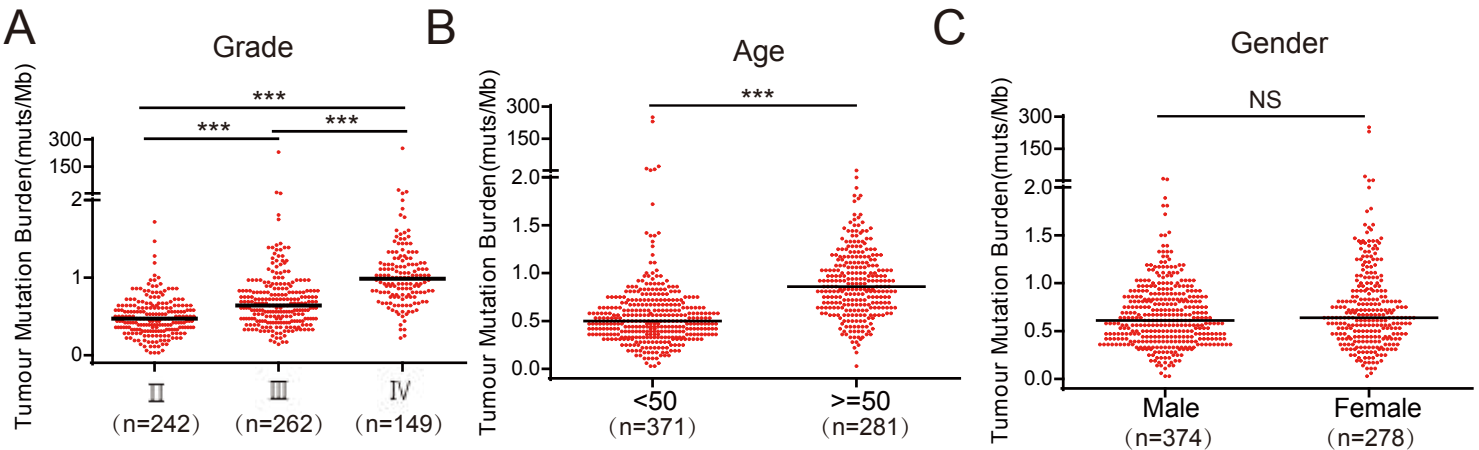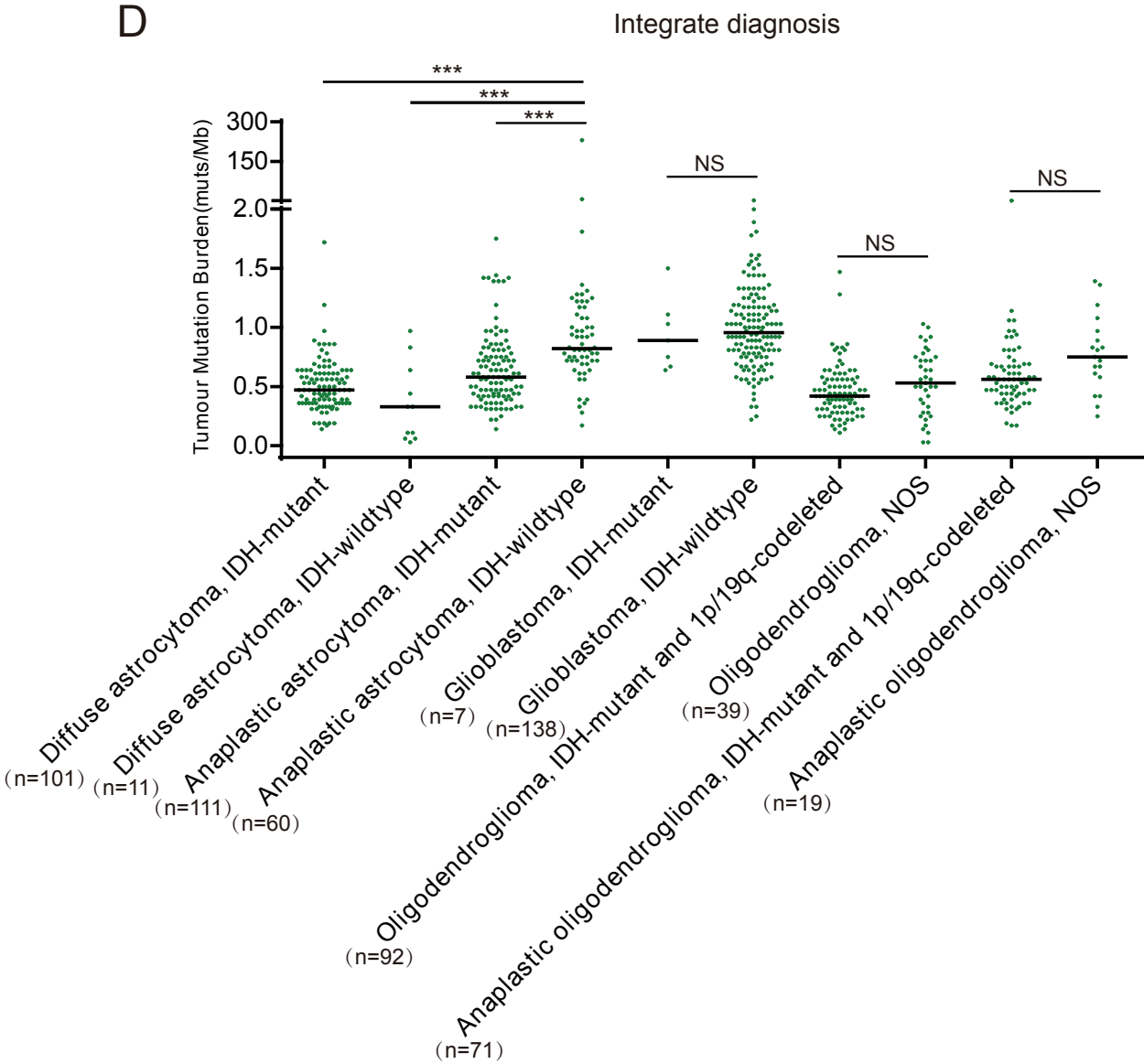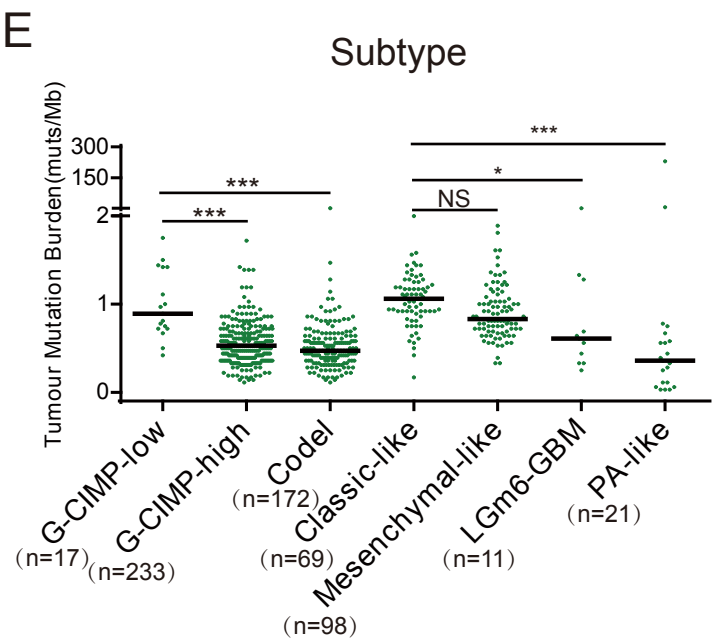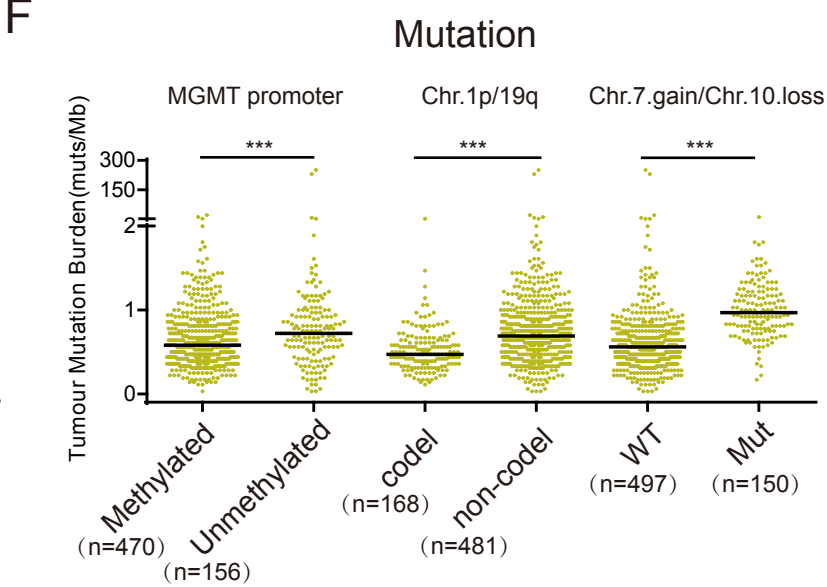

Supplement: Supplementary file 5 — Additional file 5: Supplementary Figure 3. TMB was associated with different grades (A), ages (B), sexes (C), integrated diagnoses (D), subtypes (E) and mutational statuses (F) in glioma. Statistical significance was calculated with the Mann-Whitney test for two groups and with the Kruskal-Wallis test for more than two groups. [file 12885_2020_6658_MOESM5_ESM.pdf]

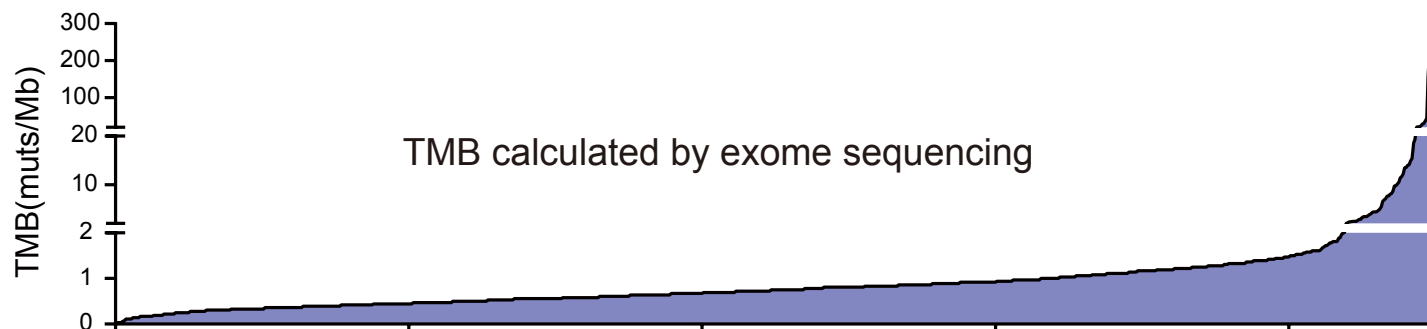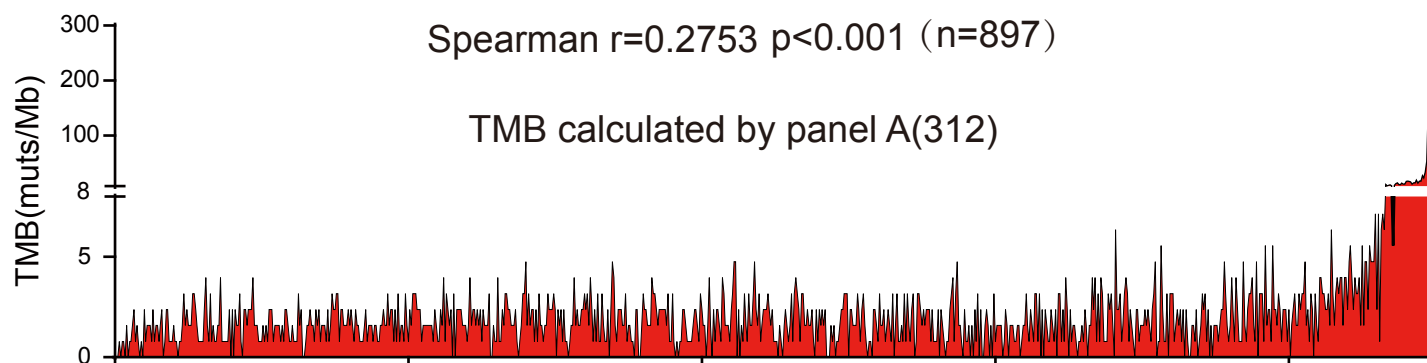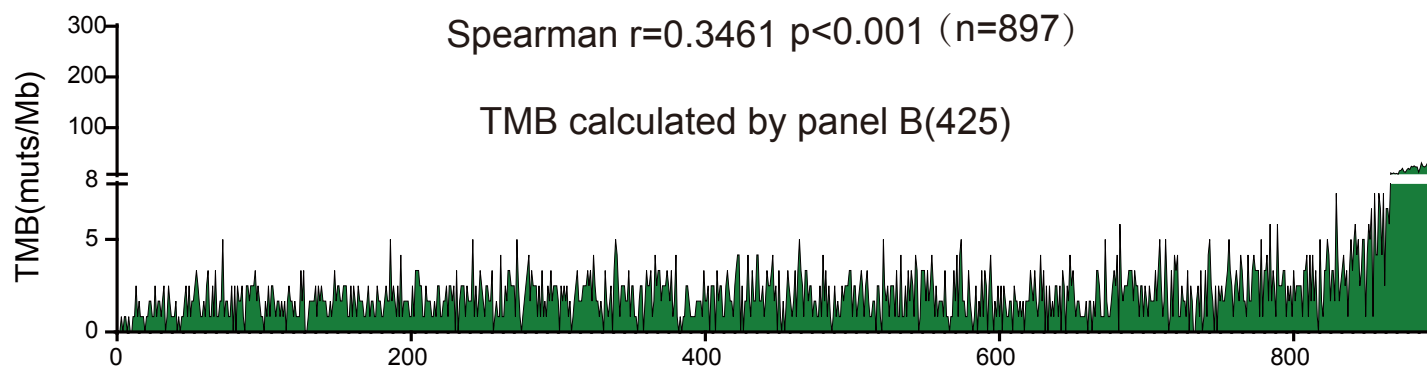

Supplement: Supplementary file 10 — Additional file 10: Supplementary Figure 5. The correlation of TMB calculated on the basis of exome sequencing and targeted genes. Gene list from two local panels used in pan-cancer analysis. Spearman’s r value and significance were calculated. [file 12885_2020_6658_MOESM10_ESM.pdf]
