# Supplementary material for: Tumor mutational burden is associated with poor outcomes in diffuse glioma
Source: BMC Cancer. 2020 Mar 12;20:213. doi: 10.1186/s12885-020-6658-1 (PMC7069200; doi:10.1186/s12885-020-6658-1)
Supplement: Supplementary file 7 — Additional file 7: Supplementary Figure 4. The transcriptional programs of the cell cycle were enriched in the TMBLow group at different ages (A), WHO grades (B), histology (C) and IDH statuses (D). The NES (normalized enrichment score), p value and FDR (false discovery rate) were calculated with GSEA software. [file 12885_2020_6658_MOESM7_ESM.pdf]

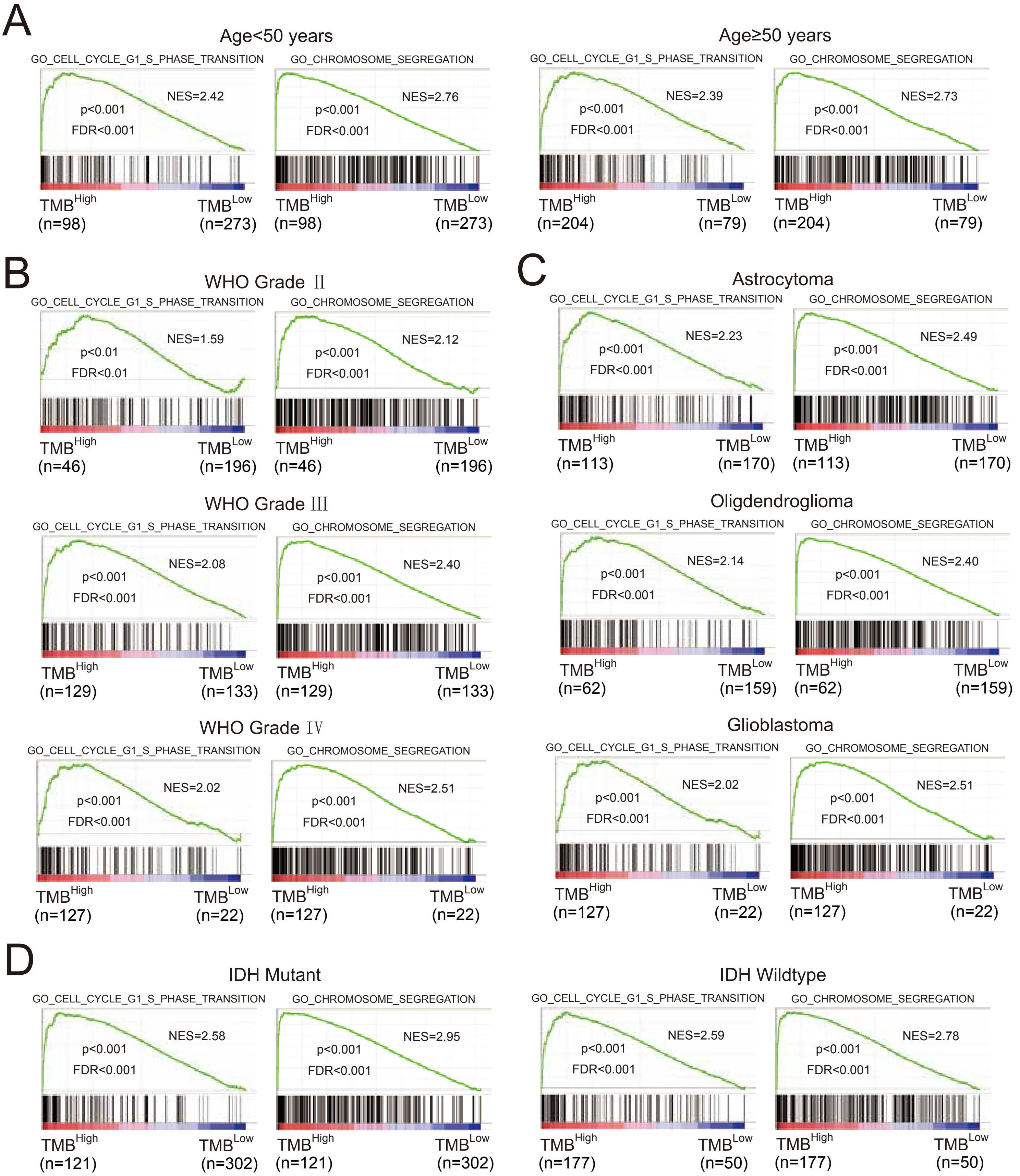

A
Age<50 years
GO_CELL_CYCLE_G1_S_PHASE_TRANSITION
NES=2.42
p<0.001
FDR<0.001
TMB High (n=98)
TMB Low (n=273)
GO_CHROMOSOME_SEGREGATION
NES=2.76
p<0.001
FDR<0.001
TMB High (n=98)
TMB Low (n=273)
Age≥50 years
GO_CELL_CYCLE_G1_S_PHASE_TRANSITION
NES=2.39
p<0.001
FDR<0.001
TMB High (n=204)
TMB Low (n=79)
GO_CHROMOSOME_SEGREGATION
NES=2.73
p<0.001
FDR<0.001
TMB High (n=204)
TMB Low (n=79)
B
WHO Grade II
GO_CELL_CYCLE_G1_S_PHASE_TRANSITION
NES=1.59
p<0.01
FDR<0.01
TMB High (n=46)
TMB Low (n=196)
GO_CHROMOSOME_SEGREGATION
NES=2.12
p<0.001
FDR<0.001
TMB High (n=46)
TMB Low (n=196)
WHO Grade III
GO_CELL_CYCLE_G1_S_PHASE_TRANSITION
NES=2.08
p<0.001
FDR<0.001
TMB High (n=129)
TMB Low (n=133)
GO_CHROMOSOME_SEGREGATION
NES=2.40
p<0.001
FDR<0.001
TMB High (n=129)
TMB Low (n=133)
WHO Grade IV
GO_CELL_CYCLE_G1_S_PHASE_TRANSITION
NES=2.02
p<0.001
FDR<0.001
TMB High (n=127)
TMB Low (n=22)
GO_CHROMOSOME_SEGREGATION
NES=2.51
p<0.001
FDR<0.001
TMB High (n=127)
TMB Low (n=22)
C
Astrocytoma
GO_CELL_CYCLE_G1_S_PHASE_TRANSITION
NES=2.23
p<0.001
FDR<0.001
TMB High (n=113)
TMB Low (n=170)
GO_CHROMOSOME_SEGREGATION
NES=2.49
p<0.001
FDR<0.001
TMB High (n=113)
TMB Low (n=170)
Oligdendroglioma
GO_CELL_CYCLE_G1_S_PHASE_TRANSITION
NES=2.14
p<0.001
FDR<0.001
TMB High (n=62)
TMB Low (n=159)
GO_CHROMOSOME_SEGREGATION
NES=2.40
p<0.001
FDR<0.001
TMB High (n=62)
TMB Low (n=159)
Glioblastoma
GO_CELL_CYCLE_G1_S_PHASE_TRANSITION
NES=2.02
p<0.001
FDR<0.001
TMB High (n=127)
TMB Low (n=22)
GO_CHROMOSOME_SEGREGATION
NES=2.51
p<0.001
FDR<0.001
TMB High (n=127)
TMB Low (n=22)
D
IDH Mutant
GO_CELL_CYCLE_G1_S_PHASE_TRANSITION
NES=2.58
p<0.001
FDR<0.001
TMB High (n=121)
TMB Low (n=302)
GO_CHROMOSOME_SEGREGATION
NES=2.95
p<0.001
FDR<0.001
TMB High (n=121)
TMB Low (n=302)
IDH Wildtype
GO_CELL_CYCLE_G1_S_PHASE_TRANSITION
NES=2.59
p<0.001
FDR<0.001
TMB High (n=177)
TMB Low (n=50)
GO_CHROMOSOME_SEGREGATION
NES=2.78
p<0.001
FDR<0.001
TMB High (n=177)
TMB Low (n=50)
